# The Human Gut Virome in Hypertension

**DOI:** 10.3389/fmicb.2018.03150

**Published:** 2018-12-19

**Authors:** Maozhen Han, Pengshuo Yang, Chaofang Zhong, Kang Ning

**Affiliations:** ^1^Key Laboratory of Molecular Biophysics of the Ministry of Education, Hubei Key Laboratory of Bioinformatics and Molecular-Imaging, Department of Bioinformatics and Systems Biology, College of Life Science and Technology, Huazhong University of Science and Technology, Wuhan, China; ^2^Department of Computer Science, City University of Hong Kong, Hong Kong, China

**Keywords:** hypertension, gut virome, virus-bacteria linkages, viral-types, diagnose

## Abstract

**Objectives:** Previous studies have reported that the gut microbiome has an important link with the development of hypertension. Though previous researches have focused on the links of gut bacteria with hypertension, little has been known about the linkage of gut viruses to hypertension and the development of hypertension, largely due to the lack of data mining tools for such investigation. In this work, we have analyzed 196 fecal metagenomic data related to hypertension aiming to profile the gut virome and link the gut virome to pre-hypertension and hypertension.

**Design:** Here, we have applied a statistically sound method for mining of gut virome data and linking gut virome to hypertension. We characterized the viral composition and bacterial composition of 196 samples, identified the viral-type of each sample and linked gut virome to hypertension.

**Results:** We stratified these 196 fecal samples into two viral-types and selected 32 viruses as the biomarkers for these groups. We found that viruses could have a superior resolution and discrimination power than bacteria for differentiation of healthy samples and pre-hypertension samples, as well as hypertension samples. Moreover, as to the co-occurrence networks linking viruses and bacteria, we found increasingly pervasive virus-bacteria linkages from healthy people to pre-hypertension people to hypertension patients.

**Conclusion:** Overall, our results have shown ample indications of the link between human gut virome and hypertension, and could help provide microbial solutions toward early diagnoses of hypertension.

## Introduction

Viruses are ubiquitous, highly abundant and diverse components of microbial communities of our bodies and natural environments (Rodriguez-Brito et al., 2010), which could shape the taxonomical and functional composition of the microbial community by altering the fitness of hosts and promoting genetic exchange (Andersson and Banfield, 2008). Previous studies have reported that the human viromes consist of viruses that infect eukaryotic cells, as well as prokaryotic cells (Handley, 2016) and they could integrate their genome in the host genome or drive continuous activation of the immune system due to chronic infection (Virgin, 2014). Furthermore, it is estimated that viral infections contribute to 15–20% of all human cancers such as prostate cancer, breast cancer, and brain cancer (McLaughlin-Drubin and Munger, 2008). It is becoming clear that viruses play a critical role in different ecosystems and it is essential to develop tools to discover and characterize more viruses, especially in human gut microbial community. Current tools for identifying virus and provirus sequences have been developed, including Phage_Finder (Fouts, 2006), Prophinder (Lima-Mendez et al., 2008), PHASTEST (Arndt et al., 2017), PhiSpy (Akhter et al., 2012), VirSorter (Roux et al., 2015), VirFinder (Ren et al., 2017), as well as general-purpose tools such as Kraken (Wood and Salzberg, 2014). Among these tools, VirSorter, VirFinder, and Kraken are the most recent programs for identifying the viral sequences in assembled metagenomics data and these tools have significantly better rates of detecting true viral contigs (Ren et al., 2017).

The human microbiome consists of more than 100 trillion microbial cells that reside mainly in the gut and form large communities (McKenna et al., 2008; Qin et al., 2010; Glasner, 2017), and the gut microbial community is an inalienable part of the host. In recent decades, the potential roles of the gut microbiome have been demonstrated, revealing that the gut microbiome has a profound influence on the immune system (Hooper et al., 2012) and the central nervous system (Sharon et al., 2016). Many links between the gut microbiome and various diseases also have been verified, including liver cirrhosis (Qin et al., 2014), type 2 diabetes (Qin et al., 2012; Wu et al., 2017), colorectal cancer (Feng et al., 2015), obesity (Ley et al., 2006; Thaiss et al., 2016) and hypertension (Li et al., 2017; Yan et al., 2017). Based on these studies, several microbial taxonomical biomarkers and functional biomarkers specific to these diseases have been discovered, and fecal microbiota transplantation is being applied as a therapeutic strategy to treat specific diseases such as inflammatory bowel disease (IBD; Anderson et al., 2012). However, most existing microbiome studies are focused on bacteria and archaea, and filter out informatics on viruses, yet viruses have been shown to be important in shaping the human microbiome and the human host (Carding et al., 2017; Nikolich-Zugich et al., 2017). Thus, the analyses of virome, a “dark matter” in the microbial community, have become critical. Among many issues associated with studying human viruses, understanding the role of the gut virome in the development of diseases is a priority.

The importance of this problem can be appreciated by considering the relationship between the virome and hypertension, which is a global public health problem and affects about 31% of the worldwide population. Previous studies have reported that primary pulmonary hypertension is associated with human immunodeficiency virus infection (Opravil et al., 1997; Barnett and Hsue, 2017) and alterations of the gut microbiota have played an essential role in the development of hypertension (Li et al., 2017; Yan et al., 2017), based on the metagenomic data from healthy individuals and hypertension patients. However, due to the limitations of identifying the viruses, the analysis of viral sequences, which have been estimated to comprise 4–17% of gut metagenomic data (Minot et al., 2011), was quite limited. Hence, a comprehensive analysis of the alterations of viruses in the gut microbiota, especially the links among healthy people, pre-hypertension people and hypertension patients, and the association between viruses and bacteria, is a priority for a more complete understanding of the underlying mechanisms for hypertension.

This study is aimed to better understand the alterations of the gut virome, especially for bacteriophages, among healthy people, pre-hypertension people, and hypertension patients, to judge whether viruses could be selected as sensitive biomarkers for diagnosing hypertension, and thus to comprehend the mechanism of the development of hypertension from a viral perspective. Hence, we selected the metagenomic data from a recent hypertension study, which reported that the novel causal role of the gut microbiota based on the metagenomic analysis of fecal samples from 196 individuals, and applied a statistically sound method for mining of gut virome data and linking viral alterations with healthy people, pre-hypertension people, and hypertension patients. This research was mainly guided by the following scientific questions: (i) How does viral diversity differ between different groups? (ii) Which viruses are enriched in different groups and can be selected to differentiate the stage of hypertension? (iii) How does the discrimination power of viruses compare to bacteria for identifying hypertension in patients? (iv) What are the virus-bacteria relationships represented by networks at different stages of hypertension?

## Materials and Methods

### Data Description

The metagenomic data used in this study were obtained from a human hypertension study (PRJEB13870) (Li et al., 2017). A total of 196 fecal samples were collected from 196 individuals of a cohort study focusing on a community from Kailuan, China, where 11 hospitals participated in the collection of these samples and conduction of the physical examinations (Li et al., 2017). Based on the systolic blood pressure (SBP) and diastolic blood pressure (DBP) information of hosts, Li et al. divided these fecal samples into three groups, namely, healthy controls (Control, SBP ≤ 125 mmHg and DBP ≤ 80 mmHg for untreated subjects, 41 samples), pre-hypertension persons (pHTN, 125 mmHg < SBP ≤ 139 mmHg or 80 mmHg < DBP ≤ 89 mmHg, subjects without antihypertensive treatments, 56 samples) and hypertension patients (HTN, 140 mmHg ≤ SBP, or 90 mmHg ≤ DBP patients without antihypertensive treatments, 99 samples) (Li et al., 2017). In this study, we downloaded the pre-filtered metagenomic data of 196 samples, which had the host genome data removed and pre-controlled the quality of these data. Based on the original information of samples, we renamed these samples and assembled the high-quality paired-end reads.

### Assembly of the Human Gut Metagenomic Data

In this study, due to the hardware limitations for metagenome assembly, to obtain more information on the virome, we randomly chose metagenomic datasets of 60 samples out of 196 samples, including 20 samples from healthy controls (C1–C20 samples), 20 samples from pre-hypertension persons (P1–P20 samples) and 20 samples from hypertension patients (H1–H20 samples), to conduct cross-assembly. MEGAHIT v1.1.1-2-g02102e1 (Li et al., 2016) was applied to cross-assemble these 60 samples using option –meta-large and with a *k*-mer list of 27, 37, 47, 57, 67, 77, 87, 97, 107, 117, and 127. To achieve a high prediction accuracy for viral sequences, contigs longer than 1,000 bp were kept for further analysis. Ultimately, we obtained 525,986 contigs, which had an average N50 length of 4,152 bp and ranged from 1,000 to 374,762 bp.

### Taxonomic Annotation and Abundance Profiling of Virus

In this work, we chose Kraken to predict viral sequences from all contigs and annotate the taxonomy of each viral sequence against its virus database (version: October 2017). These viral sequences were used to construct viral databases for calculating the viral composition of 196 fecal samples. Contig coverage (Reads Per Kilobase per Million mapped reads, RPKMs) was calculated by mapping reads to each sequence of the viral database with Bowtie2 (Langmead and Salzberg, 2012) using default settings and normalized by contig length and number of mapped reads in each sample for all 196 fecal samples. Averaged RPKM for each virus was calculated and normalized in each sample to obtain the relative abundance of each virus.

### Taxonomic Identification and Abundance Profiling of Bacteria

To obtain the bacterial composition of the 196 fecal samples, firstly, we removed the reads that mapped to viral sequences with Bowtie2 (Langmead and Salzberg, 2012) from each sample’s raw data to construct the dataset of these reads. Secondly, these datasets were used to profile the community structure of samples and bacteria information with metaphlan2 (Truong et al., 2015) using the option “–ignore_viruses,” “–ignore_eukaryotes,” and “–ignore_archaea.” Finally, the bacterial composition of samples was merged with the “merge_metaphlan_tables.py” command.

### Rarefaction Curve and α Diversity of Sample

Based on the relative abundance of viral contigs and viruses, rarefaction analyses were performed to discriminate whether we obtained the majority of the viral contigs and viruses from these fecal samples. For 196 samples, we calculated the count of shared viral contigs/viruses and all viral contigs/viruses between every two samples, and then plotted by R (version 3.4.3). To estimate the virus richness of 196 fecal samples, we calculated the α diversity using Shannon index, Simpson index, Pielou index, and the number of viruses of each sample and compared these indexes among different groups based on viral composition.

### Viral-Type Analysis Based on the Viral Composition

Similar to the enterotypes for bacteria in the microbial community, we proposed viral-types, which determined the community type of sample based on the viral composition. In this work, we analyzed and visualized the viral-type of the sample using the R packages “BiotypeR” (version: 0.1.3) and “ade4” (version: 1.7-10). Specifically, the viral-type of each fecal sample was analyzed with the PAM method using the relative abundance of viruses in each community (Arumugam et al., 2011). We calculated the Jensen–Shannon (JS) distance among samples based on the viral composition of 196 fecal samples. Silhouette index, as previously described, was applied to choose the optimal number of clusters (Lim et al., 2014; Li et al., 2017).

### Viral Biomarker Analysis

We manually checked and modified the viral taxonomy annotations to remove redundant information, and we pre-filtered the equivocal and high-level taxon. These high-quality annotations of viruses were selected to identify the biomarkers with Linear discriminate analysis (LDA) effect size (LEfSe). Specifically, the relative abundance of viruses (334 viruses) was imported into the LEfSe pipeline, and the parameters were set as follows: the alpha value for the factorial Kruskal–Wallis test (Breslow, 1970) among control, pHTN, and HTN were chosen to be 0.05. The threshold for the logarithmic LDA score for discriminative features was set at 2.0.

### Identification of pHTN and HTN Samples Based on Bacterial and Viral Composition

To identify pHTN and HTN samples, a random forest classifier was trained and tested using the random forest package (version: 4.6-12) and caret package (version: 6.0-78) in R based on viral composition and bacterial profile. In order to avoid the comparison of an uneven number of samples, we conducted rarefaction analyses of the sample groups, so that the control and pHTN groups had similar numbers of samples as the HTN group, respectively. The samples were randomly divided into training data (80%) and test data (20%). A random forest classifier was trained on training data and tested on test data for identifying pHTN and HTN patients from controls, respectively. We used a 10-fold cross-validation within the training data and the average accuracy was calculated from the 50 random forest classifiers. Finally, the performance of the model randomly selected from the 50 classifiers was measured as Area Under the Curve (AUC) when applied to test data using the pROC package (version: 1.10.0) in R.

### Virus-Bacteria Co-occurrence Network Analysis

To illustrate associations between bacteria and viruses among healthy people, pre-hypertension people, and hypertension patients, we selected the top 107 viruses and top 107 bacteria based on their relative abundances (0.1 and 0.02% was set to threshold for virus and bacteria, respectively), and focused on virus-bacteria linkages (virus-bacteria edge in the network, which is the only edge for the virus). Their relative abundance was used to calculate the associations between each bacteria and each virus using the Spearman correlation coefficient with the “cor” function in R. Likewise, all *p*-values of these associations were corrected for multiple testing using the Benjamini and Hochberg FDR-controlling procedure (Benjamini et al., 2006). Subsequently, the cutoff of the correlation and the FDR-corrected *p*-value were set at 0.5 and 0.05, respectively. The igraph package (version: 1.1.2) in R was applied to calculate the degree distribution and node betweenness, as well as the network natural connectivity. The entropy of each network was calculated based on the node degree by the entropy package (version: 1.2.1) in R. Finally, the significant associations were imported to Cytoscape (Cline et al., 2007) to visualize the viruses-bacteria bipartite networks. The nodes represent viruses and bacteria, and the edges represent the positive and negative correlations between bacteria and viruses.

## Results

### Viral Diversity of Gut Microbial Communities

**FIGURE 1:**
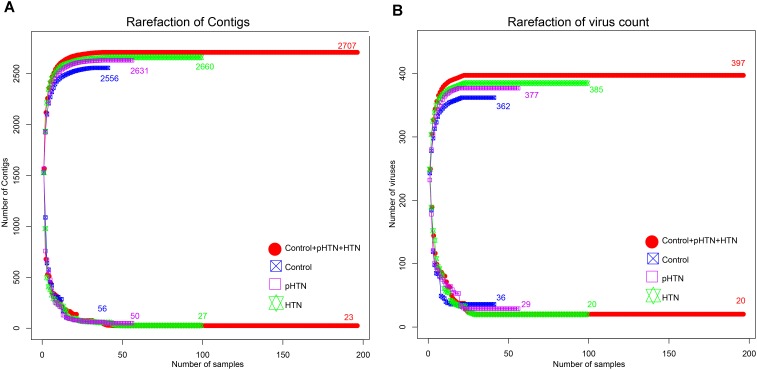
Rarefaction curves for viral contigs number and virus number in control, pHTN, HTN, and all samples. **(A)** Rarefaction curves for the number of viral contigs in control, pHTN, HTN groups, and all samples. **(B)** Rarefaction curves for the number of viruses in control, pHTN, HTN groups, and all samples. Different colors represent different samples, for example red represents all samples in control, pHTN, and HTN groups. The rising curves are accumulation curves of viral contigs and viruses in different groups. While descending curves are drawn based on the number of shared viral contigs and viruses in different groups.

**FIGURE 2:**
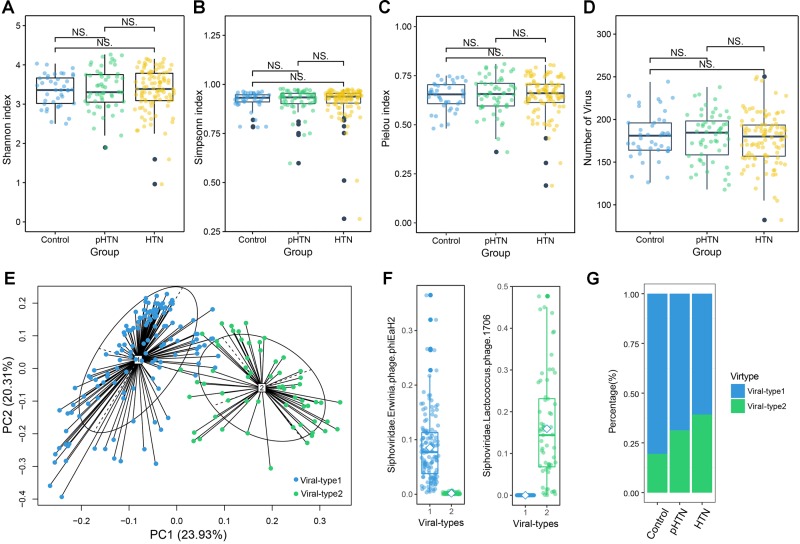
Viral diversity and the gut viral-types of gut microbial communities. Comparison of the α diversity indexes, assessing by **(A)** Shannon index, **(B)** Simpson index, **(C)** Pielou index, and **(D)** the number of viruses, based on the viral profiles in control, pHTN, and HTN groups. NS represents no significant difference. These indexes are not significantly different among these three groups (Wilcox test, all *p* > 0.05). **(E)** The planar projection and viral-types of 196 samples visualized by the PCoA. A total of 196 samples were classified into viral-type 1 (wathet) and viral-type 2 (cyan) by PCoA of Jensen–Shannon distance based on the viral composition. The most enriched virus in two viral-types is Erwinia phage phiEaH2 and Lactococcus phage 1706, respectively. **(F)** Relative abundances of the top viruses (Erwinia phage phiEaH2 and Lactococcus phage 1706) in each viral-type. **(G)** The percentage of control, pHTN, and HTN samples distributed in the two viral-types.

Based on the metagenomic assembly, we obtained 525,986 contigs. Among these contigs, 2,707 contigs were identified as viral sequences and were annotated by Kraken. Altogether, we obtained 397 viral taxa and calculated the relative abundance of these taxa. We performed the rarefaction analysis by calculating the count of shared viral contigs/viruses and all viral contigs/viruses between every two samples. The rarefaction curves based on the number of viral contigs (Figure 1A) and viruses (Figure 1B) have shown that viral contigs and virus accumulation curves among all samples approached the saturation plateau, indicating that the majority of viral contigs and viruses can be detected. We calculated the α diversity index of each sample and compared these indexes among these three groups. Specifically, we calculated the Shannon index and Simpson index based on the viral profile to compare the results of α diversity indexes, which were calculated based on bacterial genus profile in a previous study (Li et al., 2017). Additionally, we calculated the Pielou index and the number of viruses based on the viral profile, to evaluate the diversity of viruses among these groups. Unexpectedly, we found that these indexes have no significant differences among these groups (Figures 2A–D, Wilcox test, all *p* > 0.05), which is a pattern different from α diversity indexes calculated based on bacterial genera profile (which were dissimilar among groups) (Li et al., 2017).

### Gut Viral-Types

We identified the viral-types to explore differences in viral composition among the control, pHTN, and HTN groups. We obtained two viral-types suggested by the silhouette index, and the Principal Coordinate Analysis (PCoA) using Jensen–Shannon distance was applied to cluster 196 samples into two distinct viral-types (Figure 2E). We found that the most enriched bacteriophage in viral-type 1 is Erwinia phage phiEaH2 (Figure 2F), while the most enriched bacteriophage in viral-type 2 is Lactococcus phage 1706. Li et al. (2017) reported that *Prevotella* and *Bacteroides* were the most enriched genera in enterotype 1 and enterotype 2, respectively, based on the bacterial composition for the same datasets.

We found that for samples belonging to different groups, their proportions vary greatly across different viral-types and enterotypes. Most of the samples from the control (80.49%), pHTN (68.68%) and HTN groups (60.71%) were classified as viral-type 1 (Figure 2G and Supplementary Table S1), while control (73.17%), pHTN (51.88%) and HTN groups (54.55%) were classified as enterotype 2 (Li et al., 2017). This indicated that there are large differences in viral and bacterial profiles between adjacent stages (control-pHTN, pHTN-HTN).

However, compared with the enterotype of each sample identified by Li et al. (2017), we found that the enterotype and viral-type for 37 individuals were not well-matched (Supplementary Table S1), especially in pHTN (10 mismatches) and HTN stages (22 mismatches). Moreover, we found that the proportions of these mismatches in the HTN group (22.22%) and pHTN groups (17.85%) were higher than that of the control group (12.20%), indicating an increasing discrepancy between enterotype and viral-type from control to pHTN to HTN stages.

### Control, pHTN, and HTN-Enriched Viruses in the Gut Microbial Community and Biomarker Discovery

**FIGURE 3:**
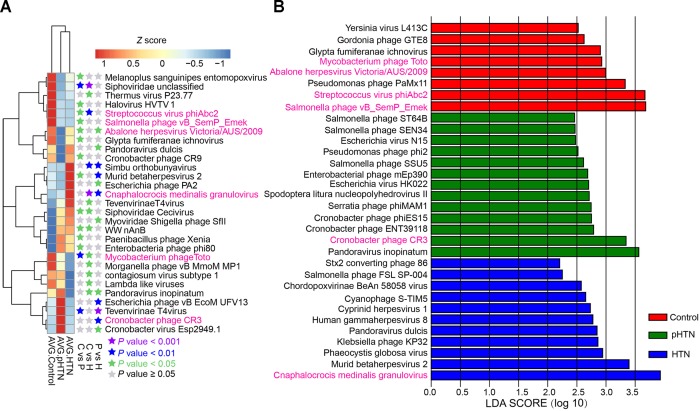
Dominant and discrepant virus analysis and biomarkers analysis for control, pHTN, and HTN groups. **(A)** Relative abundance of the top 29 most dominant and discrepant viruses among control, pHTN and, HTN groups. The threshold of *p*-value of Kruskal–Wallis test is set to be 0.05 between every two groups. AVG, average relative abundance; C, control; P, pHTN; H, HTN. The relative abundance profile was transformed to *Z* score based on the average relative abundance of corresponding viruses. **(B)** Biomarkers analysis based on viral profile for control, pHTN, and HTN groups. A total of 334 high-quality annotations of viruses were selected for biomarkers analysis by LEfSe, and 32 viruses were identified as biomarkers. The pink labeled viruses were presented in both **(A,B)**.

The enriched viruses were different in the control, pHTN, and HTN groups compared to healthy individuals. It is worth mentioning that 99 out of 397 taxa were differentially dominant in control, pHTN, and HTN groups (Kruskal test, *p* < 0.05). We selected the top 29 taxa based on the average relative abundance (more than 0.7%) and performed a cluster analysis. The cluster results suggested that the gut viral community structure of pHTN group is similar to the HTN group, rather than control group (Figure 3A). Moreover, we observed that the dominant viruses, mainly focused on bacteriophages, were different. For example, Streptococcus virus phiAbc2, Salmonella phage vB SemP Emek and Mycobacterium phage Toto were enriched in control group, and Cronobacter phage CR3 was dominant in the pHTN group, whereas *Cnaphalocrocis medinalis* granulovirus was the major contributor in the HTN group (Figure 3A) and the link between *C. medinalis* granulovirus and hypertension remains unclear.

To better distinguish samples from control, pHTN, and HTN groups, viral biomarkers were identified among the control, pHTN, and HTN groups. We further selected 334 high-quality annotations of viruses and conducted biomarkers analysis by LEfSe using the control, pHTN, and HTN groups. As a result, 32 viruses were identified as biomarkers for these groups (Figure 3B) and 22 biomarkers are bacteriophages. Specifically, eight viruses were identified as biomarkers of the control group, 13 viruses for the pHTN group, and 11 viruses for the HTN group.

### Comparison of the Discrimination Power of Viruses and Bacteria for pHTN and HTN Samples

**FIGURE 4:**
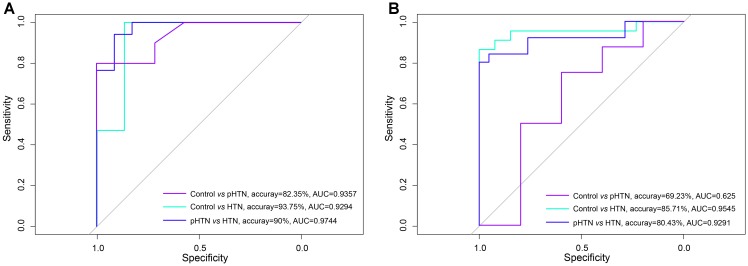
Differentiation of pHTN and HTN samples from controls. **(A)** A classification to identify pHTN and HTN samples from controls based on viral composition. **(B)** A classification to identify pHTN and HTN samples from controls based on bacterial composition. We chose a random forest classifier for every two groups and accessed by the accuracy and the AUC value. The results suggested that the viruses have a good resolution for identifying HTN samples.

Based on the viral composition and bacterial composition of the control, pHTN, and HTN groups, we investigated their discriminative power using random forest classifiers. Our results showed that based on the viral composition alone, the average accuracy for discriminating pHTN and HTN from controls was 60.88% (30.77–87.5%) and 90.02% (76.67–100%), respectively, and the average accuracy for detecting pHTN and HTN samples was 81.55% (66.67–96.67%, Figure 4A). While based on bacterial composition, the average accuracy for discriminating pHTN and HTN from controls was 75.14% (60.38–88.64%) and 81% (63.33–96.55%), respectively, and the average accuracy for detecting pHTN and HTN samples was 57.9% (30.77–80.43%, Figure 4B). These data have shown the superiority of viral composition for discrimination of samples from the pHTN, HTN, and control groups.

### Virus-Bacteria Co-occurrence Network Indicated More Targeted Virus-Bacteria Linkages in Hypertension Samples

Virus-bacteria co-occurrence network analysis was performed to reveal associations between viruses and bacteria. As for the associations of virus and bacteria within the different cohorts (control, pHTN, and HTN), we focused on virus-bacteria linkage (virus-bacteria edge in network, which is only edge for virus), as such linkages might reveal stage-specific viruses from the network perspective.

**FIGURE 5:**
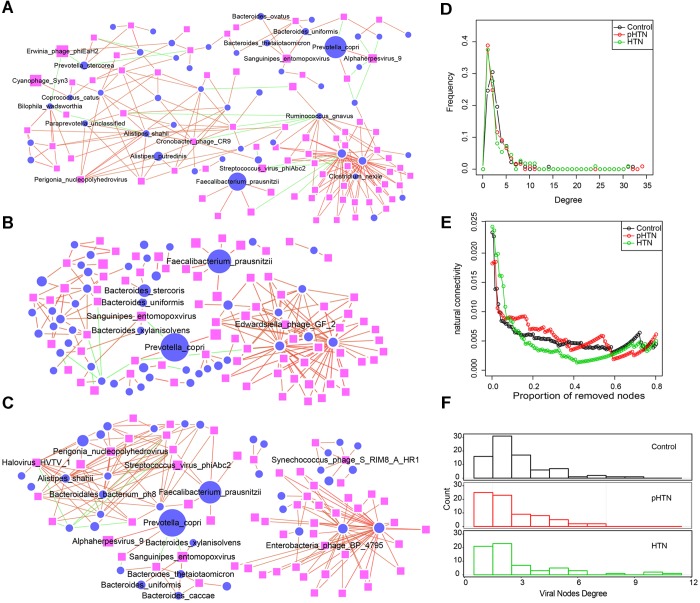
Comparison of virus-bacteria co-occurrence networks among control, pHTN, and HTN groups. Virus-bacteria co-occurrence networks for **(A)** control, **(B)** pHTN, and **(C)** HTN groups. Network nodes represent virus and species. Ellipse represents species of bacteria and rectangle represents virus. Edges represented positive associations (red) and negative associations (green) between virus and bacteria at the species level. Each node represents a bacteria or a virus and its size is proportional to the mean relative abundance within the group. The cutoff of the Spearman correlation and the FDR-corrected *p*-value were set at 0.5 and 0.05, respectively. Comparison of **(D)** degree distributions of virus-bacteria co-occurrence networks of these three groups. **(E)** Comparison of destroying degree of virus-bacteria co-occurrence network of these three groups based on the proportion of removed modes. **(F)** Comparison of differences of viral nodes degree of virus-bacteria co-occurrence network of these three groups.

Several virus-bacteria linkages have exemplified their pervasive patterns with biologically validated association. One example: many previous works have reported that Firmicutes and Bacteroidetes are the two most abundant phyla and profoundly affect human health and disease in human gut microbiota (Qin et al., 2010; Levin et al., 2017). In particular, the abundance of Firmicutes and Bacteroidetes is associated with increased blood pressure in several models of hypertension (Jose and Raj, 2015; Adnan et al., 2017). We found that three members of the Bacteroidetes, including *Bacteroides xylanisolvens, Bacteroides uniformis*, and *Bacteroides thetaiotaomicron*, had positive correlations with Sanguinipes entomopoxvirus (the enriched virus in control group, Figures 5A–C), which suggested that Sanguinipes entomopoxvirus could infect these species. The infection relationship was consistent with a previous study, which reported that the production of *ubb* gene from the *Bacteroides* genus could assist human cells to mark which proteins need to be degraded (Patrick et al., 2011). More importantly, the *ubb* gene of *Bacteroides* was obtained from Sanguinipes entomopoxvirus by horizontal gene transfer (Patrick et al., 2011). We observed that the relative abundance of Sanguinipes entomopoxvirus from controls (4.33% ± 6.15%) was higher than in the pHTN (1.94% ± 3.4%) and HTN (2.72% ± 4.26%) groups. Hence, we speculated that Sanguinipes entomopoxvirus might play a role in the development of hypertension. Another example: we also observed that *Prevotella copri* were dominant in pHTN and HTN groups, which was consistent with a previous study (Li et al., 2017), and a series of viruses, such as *Cercopithecine alphaherpesvirus 9*, were positively associated with it (Figures 5A–C) derived only from the data level.

As to the topological structure of these three virus-bacteria co-occurrence networks, we found that the pHTN network comprised 121 nodes and 181 edges (Figure 5C), while the HTN network consisted of 112 nodes and 182 edges (Figure 5C) and the control network covered 138 nodes and 225 edges (Figure 5A). The density of the pHTN and HTN network was 0.0249 and 0.0293, respectively, and the density of the control network was 0.0238. Degree distribution of the pHTN and HTN network was similar (Figure 5D). Although the network of controls comprised more nodes and edges, the network natural connectivity of the control group was more fragile than the HTN group (Figure 5E) with up to 20% nodes removed. Conversely, the network natural connectivity of control was more stable than HTN with more than 20% nodes removed (Figure 5E). Based on the degree of nodes of network, we calculated the entropy of each network, and we found that the entropy of control network (6.56) was higher than that of pHTN (6.24) and HTN (6.15), which indicated that the randomness of possible virus-bacteria interaction decreases, yet there is an increase of linkage of virus to bacteria. The number of virus-bacteria linkages (the number of viruses that only have one virus-bacteria edge in the network) increased from 16 in the control group, to 25 in the pHTN group, and 21 in the HTN groups (Figure 5F), which indicated that this linkage has become pervasive across form healthy people to pre-hypertension people and to hypertension patients.

## Discussion

Is the alteration of gut virome associated with the development of hypertension? To address this question, we studied the viral composition of fecal samples using a cohort of 196 Chinese individuals with metagenomic data, and linked the quantitative alterations of viral compositions among healthy people, pre-hypertension people and hypertension patients.

In this work, we identified 397 viral taxa, mainly consisted of bacteriophages, using Kraken and calculated their relative abundances in 196 fecal samples. The rarefaction accumulation curves calculated for all samples approached the saturation plateau, which confirmed that we have obtained most of viral contigs and viruses from these fecal samples. As to the α diversity, our results showed that the differences in richness of viruses among the control, pHTN, and HTN groups were inconspicuous, although previous work focused on the bacterial fraction of the fecal microbiome, revealed that the richness of genes and genera in the pHTN and HTN groups were reduced (Li et al., 2017).

Our results are in concordance with several previous studies. For example, we identified the viral-types of samples and compared it with enterotypes. We observed that 196 samples were divided into two viral-types and the dominated bacteriophages in viral-type 1 and viral-type 2 are Erwinia phage phiEaH2 and Lactococcus phage 1706, respectively. As to Erwinia phage phiEaH2, a previous study has reported that its relative abundance was ranked 12th among the marine viruses identified in Goseong Bay (Hwang et al., 2016). Nevertheless, to date, there are few studies on Erwinia phage phiEaH2, and without mentioning the studies on relationship between it and diseases. Although Lactococcus phage 1706 has been reported as the most abundant phage in the human gut (Roux et al., 2014), its actual role in human gut remains elusive. We found that the proportion of hypertensive samples belonging to viral-type 1 decreased among the control, pHTN, and HTN groups, while the proportions of mismatch between enterotypes and viral-types in the HTN group and pHTN group were higher than that in the control group. Hence, we speculated that the alterations of gut viral composition, especially the abundance of Erwinia phage phiEaH2 and Lactococcus phage 1706, might drive the changes of viral-type and might be associated with the development of hypertension.

A previous study has proposed that viral markers might be used to diagnose diseases and to predict treatment responses (Ludwig and Weinstein, 2005). In our work, we found that several viruses have high percentages and great variations among groups, such as Streptococcus virus phiAbc2, Cronobacter phage CR3, and *C. medinalis* granulovirus. These viruses are the first ones to be mentioned in connection with the development of hypertension and their functions in gut microbiota are still unknown. We speculate that these viruses might be involved in regulating the composition of the microbial community, further affecting the function of the microbiome, and finally influencing the development of hypertension.

We found that the average accuracies for discriminating the HTN samples from the control samples and pHTN samples based on viral composition were higher than when using bacterial composition, which suggested that viruses have a superior resolution and discrimination power for identifying HTN samples than bacteria. Although the links between viruses and hypertension remain unclear, it does not prevent us from selecting viruses as potential biomarkers for diagnosing the stages of hypertension.

As to the virus-bacteria co-occurrence network, we found that viral composition had undergone a significant change across from healthy people to pre-hypertension people and hypertension patients. Firstly, we noted an increase in the relative abundance of a number of viruses, including Lactococcus phage 1706, which might drive the change of the viral-type of sample (mainly changed from viral-type 1 to viral-type 2). Secondly, and most importantly, alterations in the viral composition led to changes in the topological structure of the virus-bacteria co-occurrence network, rendering the virus-bacteria linkage pervasive. Hence, we speculated that the alterations of gut virome might be associated with hypertension.

We have to emphasize that there is still a large gap between human gut virome and hypertension. Our study was preliminarily focused on bacteriophages and interpreted the alterations of gut virome with hypertension. One of the key findings is that even from the same batch of whole metagenome sequencing data, we have found that viruses are more advantageous than bacteria for differentiation of samples from healthy people, pre-hypertension people and hypertension patients. This has provided us with a better understanding of the relationship of microbiome (including virome) with hypertension. However, we still take it for granted that both bacteria and virus have played important roles in the development of hypertension. Thus, to fully address the role of gut virome on this development process, further study is required to completely fill the knowledge gap. For example, the development of virome database would facilitate more accurate identification and annotation of viruses from microbiome samples. More cohort studies and more omics data are needed to better understand the effects of the gut virome and gut bacteria on the development of hypertension. Finally, a series of experiments using mouse model are needed if we are to confirm the linkage between the gut virome, gut bacteria and hypertension.

## Conclusion

In this work, we obtained 397 virus taxa from 196 fecal samples and compared the viral composition among healthy people, pre-hypertension and hypertension groups. Based on viral composition, we found that there were two viral-types among these fecal samples, and viruses could be selected as biomarkers to distinguish the healthy people, pre-hypertension people and hypertension patients. Furthermore, we found that viruses have a superior resolution and better discrimination power for identifying hypertension samples than bacteria. As to the co-occurrence networks linking virus and bacteria, we found increasingly pervasive virus-bacteria linkage patterns in hypertension patients. Our results have shown ample evidence for the association of alterations in the gut virome with hypertension.

Based on these results, we suggest that viruses are potential biomarkers and could be used to diagnose hypertension at an earlier stage. In addition, our results have bridged a gap to understand the links between “virus-bacteria linkage” and disease, however, larger cohort studies are needed to completely fill the knowledge gap. In summary, our present work allows further investigation into the function of the virome on hypertension and more importantly, into our ability to diagnose and cure these diseases.

## Author Contributions

This study was designed by KN. MH collected and downloaded the data. MH, PY, and CZ analyzed the data. All authors wrote the initial draft and revised the manuscript.

## Conflict of Interest Statement

The authors declare that the research was conducted in the absence of any commercial or financial relationships that could be construed as a potential conflict of interest.

## References

[B1] Adnan S. Nelson J. W. Ajami N. J. Venna V. R. Petrosino J. F. Bryan R. M. (2017). Alterations in the gut microbiota can elicit hypertension in rats. *Physiol. Genomics* 49 96–104. 10.1152/physiolgenomics.00081.2016 28011881 PMC5336599

[B2] Akhter S. Aziz R. K. Edwards R. A. (2012). PhiSpy: a novel algorithm for finding prophages in bacterial genomes that combines similarity-and composition-based strategies. *Nucleic Acids Res.* 40:e126. 10.1093/nar/gks406 22584627 PMC3439882

[B3] Anderson J. Edney R. Whelan K. (2012). Systematic review: faecal microbiota transplantation in the management of inflammatory bowel disease. *Aliment. Pharmacol. Ther.* 36 503–516. 10.1111/j.1365-2036.2012.05220.x 22827693

[B4] Andersson A. F. Banfield J. F. (2008). Virus population dynamics and acquired virus resistance in natural microbial communities. *Science* 320 1047–1050. 10.1126/science.1157358 18497291

[B5] Arndt D. Marcu A. Liang Y. Wishart D. S. (2017). PHAST, PHASTER and PHASTEST: tools for finding prophage in bacterial genomes. *Brief. Bioinform.* 10.1093/bib/bbx121 [Epub ahead of print]. 29028989 PMC6781593

[B6] Arumugam M. Raes J. Pelletier E. Le Paslier D. Yamada T. Mende D. R. (2011). Enterotypes of the human gut microbiome. *Nature* 473 174–180. 10.1038/nature09944 21508958 PMC3728647

[B7] Barnett C. F. Hsue P. Y. (2017). HIV-associated pulmonary hypertension: a global perspective. *Adv. Pulm. Hypertens.* 15 138–143. 10.21693/1933-088X-15.3.138

[B8] Benjamini Y. Krieger A. M. Yekutieli D. (2006). Adaptive linear step-up procedures that control the false discovery rate. *Biometrika* 93 491–507. 10.1093/biomet/93.3.491

[B9] Breslow N. (1970). A generalized kruskal-wallis test for comparing K samples subject to unequal patterns of censorship. *Biometrika* 57 579–594. 10.1093/biomet/57.3.579

[B10] Carding S. Davis N. Hoyles L. (2017). The human intestinal virome in health and disease. *Aliment. Pharmacol. Ther.* 46 800–815. 10.1111/apt.14280 28869283 PMC5656937

[B11] Cline M. S. Smoot M. Cerami E. Kuchinsky A. Landys N. Workman C. (2007). Integration of biological networks and gene expression data using Cytoscape. *Nat. Protoc.* 2 2366–2382. 10.1038/nprot.2007.324 17947979 PMC3685583

[B12] Feng Q. Liang S. Jia H. Stadlmayr A. Tang L. Lan Z. (2015). Gut microbiome development along the colorectal adenoma–carcinoma sequence. *Nat. Commun.* 6:6528. 10.1038/ncomms7528 25758642

[B13] Fouts D. E. (2006). Phage_Finder: automated identification and classification of prophage regions in complete bacterial genome sequences. *Nucleic Acids Res.* 34 5839–5851. 10.1093/nar/gkl732 17062630 PMC1635311

[B14] Glasner M. E. (2017). Finding enzymes in the gut metagenome. *Science* 355 577–578. 10.1126/science.aam7446 28183934

[B15] Handley S. A. (2016). The virome: a missing component of biological interaction networks in health and disease. *Genome Med.* 8:32. 10.1186/s13073-016-0287-y 27037032 PMC4818473

[B16] Hooper L. V. Littman D. R. Macpherson A. J. (2012). Interactions between the microbiota and the immune system. *Science* 336 1268–1273. 10.1126/science.1223490 22674334 PMC4420145

[B17] Hwang J. Park S. Y. Park M. Lee S. Jo Y. Cho W. K. (2016). Metagenomic characterization of viral communities in Goseong Bay, Korea. *Ocean Sci. J.* 51 599–612. 10.1371/journal.pone.0169841 28122030 PMC5266330

[B18] Jose P. A. Raj D. (2015). Gut microbiota in hypertension. *Curr. Opin. Nephrol. Hypertens.* 24 403–409. 10.1097/MNH.0000000000000149 26125644 PMC4578629

[B19] Langmead B. Salzberg S. L. (2012). Fast gapped-read alignment with Bowtie 2. *Nat. Methods* 9 357–359. 10.1038/nmeth.1923 22388286 PMC3322381

[B20] Levin B. Huang Y. Peck S. Wei Y. Martínez-Del Campo A. Marks J. (2017). A prominent glycyl radical enzyme in human gut microbiomes metabolizes *trans*-4-hydroxy-L-proline. *Science* 355:eaai8386. 10.1126/science.aai8386 28183913 PMC5705181

[B21] Ley R. E. Turnbaugh P. J. Klein S. Gordon J. I. (2006). Microbial ecology: human gut microbes associated with obesity. *Nature* 444 1022–1023. 10.1038/4441022a 17183309

[B22] Li D. Luo R. Liu C.-M. Leung C.-M. Ting H.-F. Sadakane K. (2016). MEGAHIT v1. 0: a fast and scalable metagenome assembler driven by advanced methodologies and community practices. *Methods* 102 3–11. 10.1016/j.ymeth.2016.02.020 27012178

[B23] Li J. Zhao F. Wang Y. Chen J. Tao J. Tian G. (2017). Gut microbiota dysbiosis contributes to the development of hypertension. *Microbiome* 5:14. 10.1186/s40168-016-0222-x 28143587 PMC5286796

[B24] Lim M. Y. Rho M. Song Y.-M. Lee K. Sung J. Ko G. (2014). Stability of gut enterotypes in Korean monozygotic twins and their association with biomarkers and diet. *Sci. Rep.* 4:7348. 10.1038/srep07348 25482875 PMC4258686

[B25] Lima-Mendez G. Van Helden J. Toussaint A. Leplae R. (2008). Prophinder: a computational tool for prophage prediction in prokaryotic genomes. *Bioinformatics* 24 863–865. 10.1093/bioinformatics/btn043 18238785

[B26] Ludwig J. A. Weinstein J. N. (2005). Biomarkers in cancer staging, prognosis and treatment selection. *Nat. Rev. Cancer* 5 845–856. 10.1038/nrc1739 16239904

[B27] McKenna P. Hoffmann C. Minkah N. Aye P. P. Lackner A. Liu Z. (2008). The macaque gut microbiome in health, lentiviral infection, and chronic enterocolitis. *PLoS Pathog.* 4:e20. 10.1371/journal.ppat.0040020 18248093 PMC2222957

[B28] McLaughlin-Drubin M. E. Munger K. (2008). Viruses associated with human cancer. *Biochim. Biophys. Acta* 1782 127–150. 10.1016/j.bbadis.2007.12.005 18201576 PMC2267909

[B29] Minot S. Sinha R. Chen J. Li H. Keilbaugh S. A. Wu G. D. (2011). The human gut virome: inter-individual variation and dynamic response to diet. *Genome Res.* 21 1616–1625. 10.1101/gr.122705.111 21880779 PMC3202279

[B30] Nikolich-Zugich J. Goodrum F. Knox K. Smithey M. J. (2017). Known unknowns: how might the persistent herpesvirome shape immunity and aging? *Curr. Opin. Immunol.* 48 23–30. 10.1016/j.coi.2017.07.011 28780492 PMC5682194

[B31] Opravil M. Pechere M. Speich R. Joller-Jemelka H. I. Jenni R. Russi E. W. (1997). HIV-associated primary pulmonary hypertension. A case control study. Swiss HIV Cohort Study. *Am. J. Respir. Crit. Care Med.* 155 990–995. 10.1164/ajrccm.155.3.9117037 9117037

[B32] Patrick S. Jobling K. L. O’connor D. Thacker Z. Dryden D. T. Blakely G. W. (2011). A unique homologue of the eukaryotic protein-modifier ubiquitin present in the bacterium *Bacteroides* fragilis, a predominant resident of the human gastrointestinal tract. *Microbiology* 157 3071–3078. 10.1099/mic.0.049940-0 21885481 PMC3352274

[B33] Qin J. Li R. Raes J. Arumugam M. Burgdorf K. S. Manichanh C. (2010). A human gut microbial gene catalogue established by metagenomic sequencing. *Nature* 464 59–65. 10.1038/nature08821 20203603 PMC3779803

[B34] Qin J. Li Y. Cai Z. Li S. Zhu J. Zhang F. (2012). A metagenome-wide association study of gut microbiota in type 2 diabetes. *Nature* 490 55–60. 10.1038/nature11450 23023125

[B35] Qin N. Yang F. Li A. Prifti E. Chen Y. Shao L. (2014). Alterations of the human gut microbiome in liver cirrhosis. *Nature* 513 59–64. 10.1038/nature13568 25079328

[B36] Ren J. Ahlgren N. A. Lu Y. Y. Fuhrman J. A. Sun F. (2017). VirFinder: a novel k-mer based tool for identifying viral sequences from assembled metagenomic data. *Microbiome* 5:69. 10.1186/s40168-017-0283-5 28683828 PMC5501583

[B37] Rodriguez-Brito B. Li L. Wegley L. Furlan M. Angly F. Breitbart M. (2010). Viral and microbial community dynamics in four aquatic environments. *ISME J.* 4 739–751. 10.1038/ismej.2010.1 20147985

[B38] Roux S. Enault F. Hurwitz B. L. Sullivan M. B. (2015). VirSorter: mining viral signal from microbial genomic data. *PeerJ* 3:e985. 10.7717/peerj.985 26038737 PMC4451026

[B39] Roux S. Tournayre J. Mahul A. Debroas D. Enault F. (2014). Metavir 2: new tools for viral metagenome comparison and assembled virome analysis. *BMC Bioinformatics* 15:76. 10.1186/1471-2105-15-76 24646187 PMC4002922

[B40] Sharon G. Sampson T. R. Geschwind D. H. Mazmanian S. K. (2016). The central nervous system and the gut microbiome. *Cell* 167 915–932. 10.1016/j.cell.2016.10.027 27814521 PMC5127403

[B41] Thaiss C. A. Itav S. Rothschild D. Meijer M. T. Levy M. Moresi C. (2016). Persistent microbiome alterations modulate the rate of post-dieting weight regain. *Nature* 540 544–551. 10.1038/nature20796 27906159

[B42] Truong D. T. Franzosa E. A. Tickle T. L. Scholz M. Weingart G. Pasolli E. (2015). MetaPhlAn2 for enhanced metagenomic taxonomic profiling. *Nat. Methods* 12 902–903. 10.1038/nmeth.3589 26418763

[B43] Virgin H. W. (2014). The virome in mammalian physiology and disease. *Cell* 157 142–150. 10.1016/j.cell.2014.02.032 24679532 PMC3977141

[B44] Wood D. E. Salzberg S. L. (2014). Kraken: ultrafast metagenomic sequence classification using exact alignments. *Genome Biol.* 15:R46. 10.1186/gb-2014-15-3-r46 24580807 PMC4053813

[B45] Wu H. Esteve E. Tremaroli V. Khan M. T. Caesar R. Mannerås-Holm L. (2017). Metformin alters the gut microbiome of individuals with treatment-naive type 2 diabetes, contributing to the therapeutic effects of the drug. *Nat. Med.* 23 850–858. 10.1038/nm.4345 28530702

[B46] Yan Q. Gu Y. Li X. Yang W. Jia L. Chen C. (2017). Alterations of the gut microbiome in hypertension. *Front. Cell. Infect. Microbiol.* 7:381 10.3389/fcimb.2017.00381 PMC5573791 28884091

